# Antimicrobial susceptibility pattern of clinical isolates of *Pseudomonas aeruginosa* isolated from patients of lower respiratory tract infections

**DOI:** 10.1186/2193-1801-1-70

**Published:** 2012-12-18

**Authors:** Anab Fatima, Syed Baqir Naqvi, Sheikh Abdul Khaliq, Shaheen Perveen, Sabahat Jabeen

**Affiliations:** 1 Department of Pharmaceutics, Faculty of Pharmacy, University of Karachi, Karachi, Pakistan; 2 Department of Pharmaceutics, Faculty of Pharmacy, Hamdard University, Karachi, Pakistan; 3 Department of Pharmaceutics, Faculty of Pharmacy, Jinnah University for Women, Karachi, Pakistan

## Abstract

The present study was conducted to determine the antibiotic susceptibility pattern of *Pseudomonas aeruginosa* from sputum samples of lower respiratory tract infection patients admitted to different hospitals of Karachi. Most of the hospitals are hampered with high frequency of nosocomial infections generally caused by multiresistant nosocomial pathogen. Among Gram-negative pathogens *Pseudomonas aeruginosa* considered as most challenging pathogen. The objective of the study was to determine frequency of *Pseudomonas aeruginosa* from sputum samples and to find out susceptibility pattern against four antibiotics widely used for treatment. The sputum samples from 498 patients were collected consecutively between January 2010 and March 2011 and were cultured and identified. According to CLSI (Clinical Laboratory Standards Institute) guidelines antimicrobial susceptibility testing was performed by disc diffusion method. *Pseudomonas aeruginosa* were isolated from 24% (120/498) of the lower respiratory tract patient. A higher resistance to *Pseudomonas aeruginosa* isolate was observed with piperacillin/tazobactam and cefipime i.e. 42% and 40% respectively. Imipenem was found to be most effective antibiotic against *Pseudomonas aeruginosa* (76% sensitivity) but amikacin resistance was continuously increasing. In conclusion the frequency of *Pseudomonas aeruginosa* was also higher among lower respiratory tract infection patients with alarmingly high rate of resistance among widely used antibiotics. These findings focused on careful consideration for monitoring and optimization of antimicrobial use in order to reduce occurrence and spread of antimicrobial resistant pathogen.

## Introduction

In general practice bronchitis and pneumonia were most common lower respiratory tract infections and were related to considerable mortality and morbidity worldwide (Macfarlane et al. 2001). According to statistics 4.4% of hospital admissions and 6% of general practitioner consultations were related to lower respiratory tract infections (Anderson et al. 1993). A wide variety of antimicrobial agents with anti-pseudomonal activity along with advancement in medical and surgical care has been developed but *Pseudomonas aeruginosa* causing life threatening infections continue to cause complications in hospitals acquired infections (Mayhall 1996). *Pseudomonas aeruginosa* is a Gram-negative aerobic rod belong to family *Pseudomonadaceae*. It become considered as an opportunistic pathogens and a major cause of nosocomial infections. It was also considered as most challenging pathogen globally because of its high rate of resistance to antimicrobial agents (Hugbo and Olurinola 1992; Trilla 1994). The *Pseudomonas aeruginosa* had very minimal nutritional requirement that expedited its growth in hospital environment (Gilligan 1995). Resistance to multiple antimicrobial agents displayed by *Pseudomonas aeruginosa* and only few antibiotics found to be effective against *Pseudomonas aeruginosa* (Carmeli et al. 1999). A high resistance pattern of *Pseudomonas aeruginosa* measured as cause of higher mortality rate by *Pseudomanal* infections (Samporn et al. 2004). Different geographical locations and hospital environments showed variation in susceptibility pattern of *Pseudomonas aeruginosa* isolates therefore idiosyncrasy of isolate susceptibility pattern required for chemotherapeutic approach of *Pseudomonal* infections for better achievement of results.

In this study we aimed to determine the prevalence of *Pseudomonas aeruginosa* in lower respiractory tract infection patients and to compare their antibiotic susceptibility pattern.

## Materials and Methods

### Study setting

A total number of 498 sputum samples were collected from adult patients attended/admitted in pulmonary department of various hospitals and clinics suffered from lower respiratory tract infections in 14 months period from January 2010 to March 2011. Out of 498 samples 120 samples were *Pseudomonas aeruginosa*.

### Microbiology/sample processing

The samples were transferred to microbiology laboratory and were analyzed within 30 min to 1 hour of collection. Nutrient agar, MacConkey agar and blood agar (Oxoid UK) used for streaking of sample and then incubated at 37°C for 24 hours as described by chessborough (Cheesborough 2002). After incubation *Pseudomonas* isolation agar media (Oxoid UK) used for sub-culturing of isolate obtained. The pure isolates of *Pseudomonas aeruginosa* were transferred to 1% nutrient agar slant and stored in the refrigerator at 4 ± 1°C. Diifferent identification tests were performed on suspected *Pseudomonas aeruginosa* and were characterized and identified i.e. Gram-stain, colonial morphology, positive oxidase reaction, production of pyocyanin on Mueller-Hinton agar (Oxoid UK), citrate utilization and growth at 42°C.

### Antibiotic susceptibility test

By disk diffusion technique antibiotic susceptibility pattern of isolates on commonly used antibiotics was performed on Mueller-Hinton agar medium according to Clinical Laboratory Standard Institute (CLSI) guidelines (NCCLS 1995). Paper disk were impregnated with antibiotics (Sigma chemicals): imipenem (10 μg), Amikacin (30 μg), piperacillin / tazobactam (100/10 μg) and cefipime (30 μg) respectively and incubated at 37°C for 24 hours in 5–10% CO_2_ enriched environment. The medium containing antibiotic disks were quality controlled daily by standard culture. After defined incubation period the diameter of zone of inhibition was measured and interpretation of result based on CLSI guidelines was performed (NCCLS 1995).

## Result

**Figure 1:**
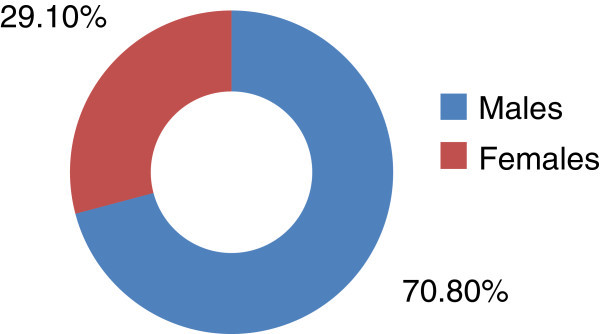
**% occurrence of lower respiratory tract infections in males and females by Pseudomonas aeruginosa.**

In 14 months period a total of 498 sputum specimens were collected consecutively from different hospitals of Karachi and a total of 120 *Pseudomonas aeruginosa* strains (24%) were isolated of which 85 samples (70.8%) and 35 samples (29.1%) were reported from males and females respectively (Figure 1).

**Figure 2:**
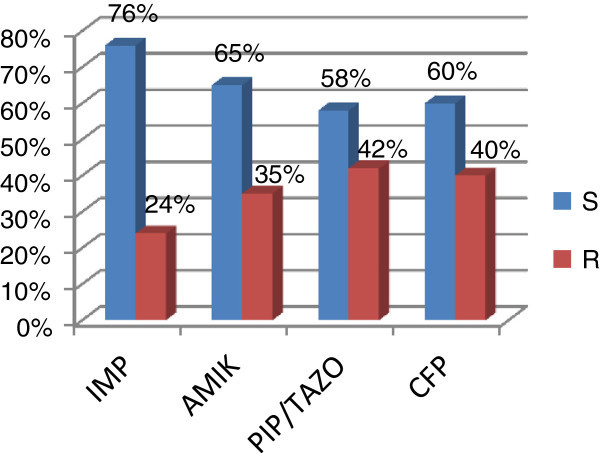
**Resistance rate of Pseudomonas aeruginosa.** IMP: imipenem, AMIK: amikacin, PIP/TAZO: piperacillin/tazobactam, CFP: cefipime.

The antibiotic resistance pattern of isolates presented in Figure 2. The most effective antibiotic was from Carbapenem i.e. imipenem and its resistance rate was detected as 24%.

Piperacillin/tazobactam and cefipime showed more resistance i.e. 42% and 40% respectively. Amikacin also showed 35% resistance. Graphically Figure 2 clearly reflect antibiotic susceptibility profile of *Pseudomonas aeruginosa* isolates and showed that imipenem was most sensitive then all other antibiotics used.

## Discussion

The main objective of this study was to investigate epidemiological data of *Pseudomonas aeruginosa* strains in lower respiratory tract infection patients and to determine the antimicrobial resistance pattern of bacteria against some commonly used antibiotics. The predominance of *Pseudomonas aeruginosa* resistance considered as serious problem in many countries (Agarwal et al. 2006; Ako-Nai et al. 2006; Balkhy et al. 2006; Lizioli et al. 2003). It was also reported that *Pseudomonas aeruginosa* is one of the most common nosocomial pathogen and a leading cause of nosocomial respiratory tract infection (Gilligan 1995; Jarvis and Martone 1992; Jarlier et al. 1996).

From previous literature high rate of resistance against carbapenem, quinolones and third generation cephalosporin had been detected *in Pseudomonas aeruginosa* (Hancock 1998; Quinn 1998; Sader et al. 1998). In our study resistance rate against imipenem from carbapenem group was determined as 24% which was considerably accelerating towards higher side in Pakistan and this was in agreement with previous study conducted by Akhtar N which showed resistance rate of 26.1% (Akhtar 2010). It was reported that resistance to imipenem was 14% in Spain (Bouza et al. 1999), 19.3% in Italy (Bonfiglio et al. 1998) and was 68% in Saudi Arabia (Rotimi et al. 1998).

In a previous hospital study resistance rate among *Pseudomonas aeruginosa* was only 5–9% against amikacin (Bouza et al. 1999; Gerding et al. 1991). In the present study the rate of amikacin resistance was found to be relatively high i.e. 35% however it may in accordance with Friedland et al. who reported that amikacin resistance related to more intensive usage of amino glycosides (Friedland et al. 1992). The increasing rate of amikacin in Pakistan was also reported by Akhtar N i.e. 21.3 against *Pseudomonas aeruginosa* (Akhtar 2010). It was further investigated that when amikacin used in combination with impenem i.e. as combination therapy resistance rate among *Pseudomonas aeruginosa* was reduced to 10% (Bustamante et al. 1987).

Piperacillin/tazobactam resistance rate was 7% in a nationwide in Spain (Bouza et al. 1999). Contrary to this resistance rate in isolates was higher in our study (42%). In a previous study equivalent safety and efficacy of intravenous Piperacillin/tazobactam with intravenous imipenem/cilastatin was reported for intra-abdominal infections caused by *Pseudomonas aeruginosa* but presently resistance was different for both antibiotics which might be due to extensive usage of this combination in our hospitals as life saving antibiotics (Erasmo et al. 2004).

In the present study resistance rate of *Pseudomonas aeruginosa* against cefepime observed was deviated from reported data as it showed 40% resistance while in previous reported data it did not exceed 17% (Bouza et al. 1999).

The data obtained from our study in Pakistan it was probable that application of conventional agents for the empirical treatment become complicated by the respiratory pathogens with accelerating resistance to antibiotics. Therefore for an effective management of lower respiratory tract infection an ultimate and detailed bacteriological diagnosis and susceptibility testing required to overcome global problem of antibiotic resistance and by encouraging greater understanding of this problem different solutions can be planned by health care providers.
